# Barriers and Facilitators for Implementing a Decision Support System to Prevent and Treat Disease-Related Malnutrition in a Hospital Setting: Qualitative Study

**DOI:** 10.2196/11890

**Published:** 2019-05-09

**Authors:** Mari Mohn Paulsen, Cecilie Varsi, Ingvild Paur, Randi Julie Tangvik, Lene Frost Andersen

**Affiliations:** 1 National Advisory Unit on Disease-related Malnutrition Department of Cancer Medicine Oslo University Hospital Oslo Norway; 2 Institute of Basic Medical Sciences Department of Nutrition University of Oslo Oslo Norway; 3 Center for Shared Decision Making and Collaborative Care Research, Division of Medicine Oslo University Hospital Oslo Norway; 4 Department of Clinical Medicine Faculty of Medicine University of Bergen Bergen Norway

**Keywords:** malnutrition, implementation science, eHealth, qualitative research, decision support systems, clinical

## Abstract

**Background:**

Disease-related malnutrition is a challenge among hospitalized patients. Despite guidelines and recommendations for prevention and treatment, the condition continues to be prevalent. The MyFood system is a recently developed decision support system to prevent and treat disease-related malnutrition.

**Objective:**

To investigate the possible implementation of the MyFood system in clinical practice, the aims of the study were (1) to identify current practice, routines, barriers, and facilitators of nutritional care; (2) to identify potential barriers and facilitators for the use of MyFood; and (3) to identify the key aspects of an implementation plan.

**Methods:**

A qualitative study was performed among nurses, physicians, registered dietitians, and middle managers in 2 departments in a university hospital in Norway. Focus group discussions and semistructured interviews were used to collect data. The Consolidated Framework for Implementation Research (CFIR) was used to create the interview guide and analyze the results. The transcripts were analyzed using a thematic analysis.

**Results:**

A total of 27 health care professionals participated in the interviews and focus groups, including nurses (n=20), physicians (n=2), registered dietitians (n=2), and middle managers (n=3). The data were analyzed within 22 of the 39 CFIR constructs. Using the 5 CFIR domains as themes, we obtained the following results: (1) Intervention characteristics: MyFood was perceived to have a relative advantage of being more trustworthy, systematic, and motivational and providing increased awareness of nutritional treatment compared with the current practice. Its lack of communication with the existing digital systems was perceived as a potential barrier; (2) Outer settings: patients from different cultural backgrounds with language barriers and of older age were potential barriers for the use of the MyFood system; (3) Inner settings: no culture for specific routines or systems related to nutritional care existed in the departments. However, tension for change regarding screening for malnutrition risk, monitoring and nutritional treatment was highlighted in all categories of interviewees; (4) Characteristics of the individuals: positive attitudes toward MyFood were present among the majority of the interviewees, and they expressed self-efficacy toward the perceived use of MyFood; (5) Process: providing sufficient information to everyone in the department was highlighted as key to the success of the implementation. The involvement of opinion leaders, implementation leaders, and champions was also suggested for the implementation plan.

**Conclusions:**

This study identified several challenges in the nutritional care of hospitalized patients at risk of malnutrition and deviations from recommendations and guidelines. The MyFood system was perceived as being more precise, trustworthy, and motivational than the current practice. However, several potential barriers were identified. The assessment of the current situation and the identification of perceived barriers and facilitators will be used in planning an implementation and effect study, including the creation of an implementation plan.

## Introduction

Disease-related malnutrition is a challenge in hospitals, with 30% to 50% of patients being malnourished or at risk for malnutrition [1-5]. The condition leads to higher morbidity and mortality rates among patients [5-8] and longer length of stay [6,9,10]. This generates increased economic costs for the health care sector [7,10,11]. According to Norwegian [12] and European [13] guidelines, all patients at malnutrition risk should have an individualized nutrition care plan, including documentation of nutritional status, needs, dietary intake, and recommended treatment. The reported barriers to adequate nutritional care for hospitalized malnourished patients include the absence of routines [14,15], lack of knowledge, assignment of responsibility [16], and lack of skills and tools to estimate individual dietary needs and the energy and protein content in hospital food [14,17].

Studies have shown that hospitals can benefit from implementing technology to identify, handle, and follow up with patients at risk of malnutrition. Digital tools and apps may reduce the workload of health care professionals and the time spent for nutritional assessment [18].

**Figure 1:**
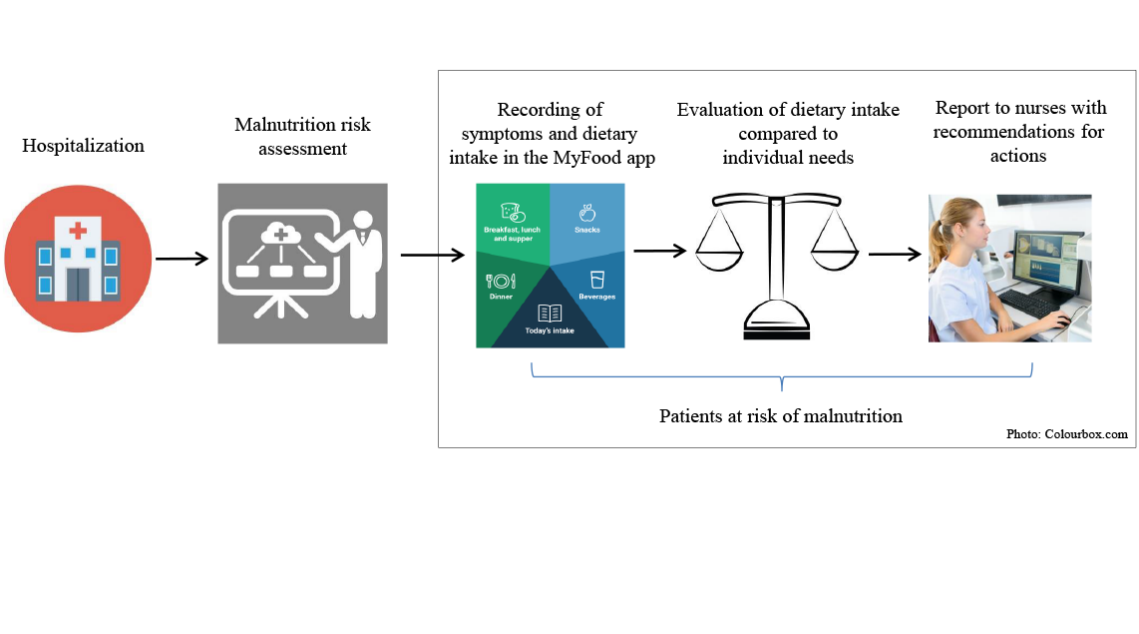
Patient flow from hospitalization, identification of malnutrition risk, and use of the MyFood system.

We developed the *MyFood* tool, a decision support system for use among hospitalized patients at risk of malnutrition. The MyFood system includes an app for tablets and a website. Figure 1 shows the intended use of the MyFood system.

A consistent finding in clinical and health services research is the failure to translate evidence into practice [19]. The implementation of electronic health (eHealth) interventions is often challenging, with many failing to demonstrate predicted benefits [20]. For implementation to succeed, it is recommended that the readiness for implementation be assessed and the barriers and facilitators be identified in advance [21]. Theoretical frameworks may guide this assessment. The Consolidated Framework for Implementation Research (CFIR) [22] is widely used to identify barriers and facilitators [23-26].

To obtain a better understanding of how to implement the MyFood system in clinical hospital practice and to be able to create an implementation plan, we performed a qualitative study among health care professionals. The specific aims were (1) to identify current practice, routines, barriers, and facilitators for nutritional care; (2) to identify potential barriers and facilitators for the use of a decision support system (MyFood); and (3) to identify the key factors for an implementation plan.

## Methods

This study is part of a research project involving the development and evaluation of a decision support system to prevent and treat disease-related malnutrition as a proof of concept. The MyFood intervention will be implemented in the hospital departments in a randomized controlled trial after this study is completed.

### The MyFood System

The MyFood system is developed in response to an identified need for better tools to follow up with patients who suffer from disease-related malnutrition. The functions and content of the tool are based on the Norwegian guidelines for prevention and treatment of disease-related malnutrition [12], the Norwegian Directorate of Health recommendations on nutrition in health and care services [27], and the recommended tasks included in the focus area of disease-related malnutrition in the Norwegian Patient Safety Program [28]. According to the patient safety program, 4 tasks are necessary to prevent and treat disease-related malnutrition in hospitals: (1) screening for risk of malnutrition; (2) dietary assessment; (3) nutritional treatment; and (4) documentation [28]. Hence, MyFood does not provide new tasks for health care professionals but intends to provide a system to perform and follow the guidelines and recommendations available.

**Figure 2:**
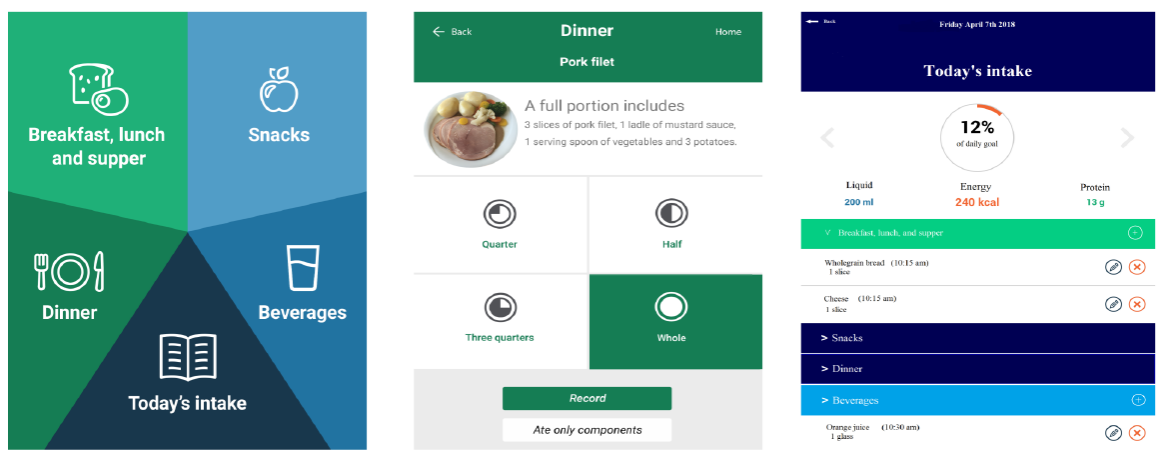
Dietary assessment in the MyFood app and evaluation of dietary intake compared with individual needs.

The MyFood system consists of 4 modules: module 1, collection of information about the patient (body weight, height, nutrition-related symptoms, nutritional situation, and allergies); module 2, dietary assessment function; module 3, evaluation of recorded dietary intake compared with individual needs for energy, protein, and liquids; and module 4, report function, including recommendations for nutrition-related actions tailored to the individual patient and a template for a nutrition care plan. Figure 2 illustrates the dietary assessment and evaluation functions (modules 2 and 3) of the app. The patients record their daily dietary intake in the app. If the patient is unable to record, the nurses perform the recording on behalf of the patient. Both patients and health care professionals may keep track of the evaluation in module 3. The development of the MyFood app (modules 1 to 3) and evaluation of the dietary assessment function are described in a previous study [29]. The report function (module 4) is intended for use by nurses or other health care professionals to monitor and follow up on a patient’s nutritional status and treatment. Module 4 is a website where the nurses gain access and retrieve information about patients by logging into the system.

The MyFood system was externally developed in cooperation with selected hospital departments. The managers of the hospital departments were involved in the structural issues and facilitated the research project. Nurses, registered dietitians, and patients participated in the development of the design, content, usability, and functionality [29].

### Study Design and Participants

We conducted a qualitative study among health care professionals from 2 departments at a university hospital in Norway. The data collection period was January to February 2018. The study was based on 4 focus group discussions and 7 individual interviews. The health care professionals were purposively selected for the focus group discussions and interviews.

The study was performed in accordance with the Helsinki declaration and was acknowledged by the Norwegian Regional Ethical Committee (2016/1464). Written informed consent was obtained from all participants.

### The Consolidated Framework for Implementation Research Framework as a Basis for the Interview Guide

The CFIR is a compilation of 39 constructs related to implementation and divided into 5 domains: characteristics of the intervention, outer setting, inner setting, characteristics of the individuals involved, and the process of implementation. These constructs can be considered when identifying local barriers to implementation [22]. According to Damschroder et al [22], researchers may select the constructs from the CFIR that are most relevant for their study setting. In this study, the 39 constructs of the CFIR [22] were explored and used to develop a semistructured interview guide. A total of 13 constructs were considered relevant for the context, and open-ended questions based on these were included. The interview guide was adapted for different groups of health care professionals to adjust for relevant differences in roles or tasks. For example, the construct regarding structural characteristics in the outer setting domain was only addressed to middle managers. Not all the CFIR constructs were considered relevant. For example, several of the constructs related to the process and the outer setting domains were not included. This study was a preimplementation study, and at this stage, we were most interested in the local factors in the 2 hospital departments to be able to set the performance goals of an implementation and effect study and to develop an implementation plan. The interview guide included questions about the organization and the routines related to the food and nutritional care of the patients, including responsibility, management commitment, and challenges. Perceived barriers and facilitators for the use of the MyFood tool and for performing an intervention study in the departments were also included in the guide. During the focus group discussions and interviews, the MyFood app was demonstrated for the health care professionals.

### Focus Group and Interview Procedure

The focus group discussions and individual interviews were conducted by the first author in a meeting room in the hospital department or at the interviewee’s office. A secretary assisted the first author during the focus groups. The focus group discussions were 45 to 55 min long, and the individual interviews were 30 to 50 min long. Focus groups facilitate communication between participants [30] and were chosen as the method for nurses because they are engaged in the daily care of patients. Each focus group included 4 to 7 nurses. The first focus group discussion served as a pilot to test the interview guide. After the focus group discussion, the interviewees were asked for feedback on the structure and phrasing of questions as well as the focus group situation. The pilot focus group did not result in any fundamental changes to the interview guide and was therefore included in the main analysis. Individual interviews were performed among the middle managers, physicians, and registered dietitians for feasibility reasons.

The focus group discussions and the individual interviews were recorded with a digital voice recorder (Olympus WS-853). A dictaphone app developed by the University Center for Information Technology at the University of Oslo (UiO) [31] was used as a backup. In addition, notes were taken immediately after each focus group and interview. The audio recordings were transcribed verbatim using the software f4transkript (Marburg).

### Analysis

**Figure 3:**
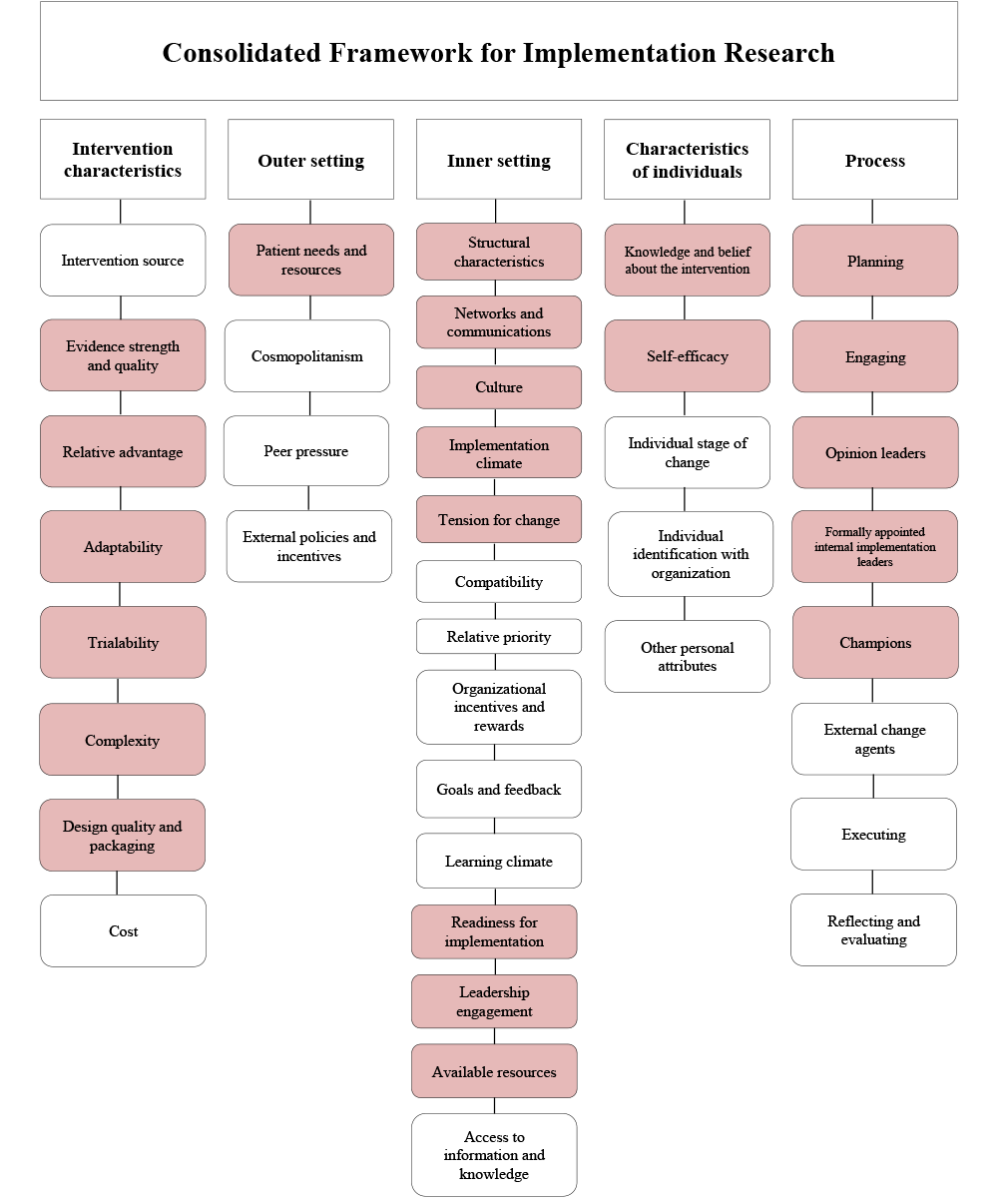
Overview of the Consolidated Framework for Implementation Research. The analyzed data were sorted into 22 constructs (red boxes) for the assessment of current practices and the identification of barriers and facilitators. Data for the remaining constructs (white boxes) could not be obtained.

The transcripts and notes were analyzed using a thematic analysis in a stepwise manner as described by Braun and Clarke [32], using a deductive approach. The transcripts were analyzed using NVivo version 11 (QSR International). The first step in the analysis was to read through all the transcripts and take notes to obtain an overall understanding of the material. Second, initial codes were created as nodes based on the 5 domains in the CFIR framework and subnodes for the 39 CFIR constructs [22]. Some parts of the transcripts did not directly fit into any of the CFIR constructs, and in these cases, new codes were created. Phase 3 involved searching for themes. As we followed a deductive approach, based on the domains and constructs in the CFIR framework, the primary task here involved resorting and reevaluating the codes. The final step was the review process. The codes that did not fit into the CFIR framework were particularly evaluated and reconsidered. If they were found relevant, they were included in the current constructs. A total of 22 CFIR constructs were included in the analysis (Figure 3). The results described for the 22 CFIR constructs were reviewed and have been elaborated with regard to the specific study aims in the Discussion section.

To enhance trustworthiness [33], including credibility, confirmability, dependability, and transferability [34], the results were analyzed systematically in a stepwise manner. This included the following: involving all authors in the development of the interview guide and involving the first (MMP) and second (CV) authors in the development of the coding categories and the interpretation of the results; including different health care professionals in the interviews; and audio taping and transcribing the material verbatim.

## Results

### Demographics

The focus group discussions included 20 nurses, with a mean age of 30 years and a range of 24 to 39 years. Table 1 shows the characteristics of the nurses in the 4 focus group discussions.

The individual interviews included 2 physicians, 2 registered dietitians, and 3 middle managers. They were all female with a mean age of 39 years, ranging from 27 to 45 years.

**Table 1:** Characteristics of the nurses in the 4 focus group discussions.

Characteristic		FGD^a^ 1 (n=4)	FGD 2 (n=4)	FGD 3 (n=7)	FGD 4 (n=5)
**Gender (n)**					
	Male	1	1	0	0
	Female	3	3	7	5
**Age (years)**					
	Mean	32	27	29	31
	Range	26-39	26-30	25-38	25-36
**Experience (n)**					
	<4 years	0	3	2	1
	≥4 years	4	1	5	4

^a^FGD: focus group discussion.

**Table 2:** Potential barriers and facilitators for use of the MyFood system, identified in stakeholder focus group discussions and semistructured interviews.

CFIR^a^ domain	Barriers	Facilitators
Intervention characteristics	Lack of automatic transfer to the electronic patient record; Hygienic aspects of using tablet computers among the patients; Potentially demotivational for patients who strive to meet their dietary needs	More trustworthy, systematic, fun, and easy to use than the current practice; May increase awareness on nutritional care and treatment; Positive attitudes among health care providers to test the MyFood tool in an intervention study; Intuitive, neat, and user-friendly design
Outer setting	Lack of current routines for screening for malnutrition risk; Nurses’ perceptions of nagging patients regarding food intake; Different cultural backgrounds among patients; Language barriers among non-native patients; Patients fasting before surgery or medical examinations; Elderly patients not familiar with tablet computers	Potentially earlier implementation of nutritional treatment among the patients; Empowerment of patients in the recording of dietary intake
Inner setting	Ambiguity among health care providers who have the primary responsibility for nutritional care and treatment; Prejudices among some physicians regarding the role of nutrition in the treatment process; Diverging focus between different health care providers, which may confuse the patients; Lack of culture and specific routines for nutritional care; Weak foundation on nutritional care among management; Limited availability of computers to use the MyFood report function; Limited available time	High stability in the departments’ staff of health care professionals; Good cooperation between health care professionals; Assumptions among nurses regarding the importance of nutrition; Desire among nurses for better tools for dietary assessment and follow-up; Potentially time saving if nurses do not have to do manual calculations of dietary intake themselves
Individual characteristics	—^b^	Perceived self-efficacy among nurses in the ability to use the MyFood tool.

^a^CFIR: Consolidated Framework for Implementation Research.

^b^Not applicable.

### Identification of Barriers and Facilitators Using the Consolidated Framework for Implementation Research

The current practice with nutritional care, perceived barriers and facilitators for the use of the MyFood system, and the identified key aspects to include in an implementation plan are presented according to the 5 domains of the CFIR framework and subdivided into the relevant constructs (Figure 3).

The perceived barriers and facilitators for use of the MyFood system are summarized in Table 2.

### Intervention Characteristics

Evidence strength and quality relates to stakeholders’ perceptions of the quality and validity of evidence supporting the belief that the intervention will have desired outcomes [22]. The interviewees acknowledged that the evidence-based recommendations forming the basis of the MyFood system were known and accepted. They claimed that several of the functions in the MyFood tool were already performed at the hospital departments, although in a more unstructured manner:

I think this is kind of the same, but gathered more in one place. And this [MyFood] provides a better overview.

Registered dietitian

Relative advantage is the stakeholders’ perceptions of the advantage of implementing the intervention versus an alternative solution [22]. Most of the interviewees perceived the dietary assessment function in MyFood as easier, more trustworthy, systematic, and precise compared with the paper-based dietary assessment forms currently in use. They also reported that MyFood could increase awareness of nutritional deficiencies and lead to the implementation of nutritional treatment at an earlier stage:

I think it’s easier when you can trust it. [...] Then the physician will trust it more I think, that this is actually correct recorded, this is exactly what was eaten [...] Compared to using a form that you don’t know is complete. Then it’s easier to take action if you trust the recording. I think.

Nurse

The health care professionals’ perceptions of the dietary recording in the app were that it would be more fun and motivational than traditional paper recordings and that the tool was better suited for the future:

You know, we are not spoiled with new, fun technical solutions in the healthcare system. So most of us think it’s fun when something new arrives. Because it’s fun to have a gadget, you know. I think people would suddenly regard it as fun to record food, compared to that form [the paper-based dietary recording form] for which you need to scratch your head to guess the calorie intake.

Nurse

Adaptability relates to the degree to which an intervention can be adapted, tailored, refined, or reinvented to meet local needs [22]. The respondents gave feedback on how they perceived MyFood could fit into their existing work practice. A potential barrier was that MyFood does not communicate with the electronic patient record (EPR), which means that the health care professionals need to copy the information from the MyFood website and paste it into the EPR. However, suggestions for how to overcome this issue were proposed:

It’s quite okay because we try to become paperless. And if we can just copy from that [MyFood] to the electronic journal. The dietary paper forms [paper-based dietary assessment forms used today] easily gets lost. This is like... It seems more secure.

Nurse

The hygienic aspects of using tablet computers among the patients were discussed, including patients with special considerations regarding infections. Several solutions for getting around this issue were suggested, for example, using a cover or plastic bag around the tablet computer.

Trialability is defined as the ability to test the intervention on a small scale in the organization and to be able to reverse course if warranted [22]. Attitudes to being part of an intervention study to test the MyFood system were positive. All groups of respondents reported being used to participate in clinical trials owing to having an ongoing study in the department at almost any time.

The complexity construct describes the perceived difficulty of implementation, reflected by duration, scope, radicalness, disruptiveness, centrality, intricacy, and the number of steps required for implementation [22]. MyFood was perceived as easy to use and navigate. None of the interviewees reported that the tool seemed complex:

I’m technically retarded and even I think this seems okay.

Nurse

Design quality and packaging are defined as the perceived excellence in how the intervention is bundled, presented, and assembled [22]. The layout of the MyFood system was described by health care professionals as having a user-friendly, intuitive, and neat design. The possibility to record only the components of a dish, in addition to the proportion of portion size (Figure 2), was highlighted as an advantage. However, a few nurses mentioned that the illustration of the percent of achievement of energy, protein, and liquid intake compared with individual needs (Figure 2) could potentially be demotivational for some patients:

When you have only eaten 10% of your need, and feel that you have eaten a lot and that you’ll never be able to achieve your goal.

Nurse

### Outer Setting

The patient needs and resources construct concerns the extent to which patient needs, as well as barriers and facilitators to meet those needs, are accurately known and prioritized by the organization [22]. The health care professionals elaborated on current practices and whether screening for malnutrition risk was performed. Some routines with screening existed in one of the departments. In the other department, there were preconceptions that few of the patients eat and, therefore, they did not conduct routine malnutrition risk screening:

We haven’t done that [screening for malnutrition] until now. We really expect that no one eats. We expect that they either become nauseous or the food tastes strange, after quite a short time. And we expect that everyone at some time point will start with TPN [total parenteral nutrition]. So I think we aren’t good enough to..., you know, we know that we’ll get there [TPN] in 4 days anyway, so there’s no point to keep it going with ordinary food.

Nurse

The experience of nagging patients about food intake was highlighted by several nurses, middle managers, and registered dietitians:

[...] They [the patients] think we are whining too much about the food because they don’t regard it as very important. You know, sick, reduced appetite, and all that...

Nurse

However, some patients were described as being very motivated and perceived the achievement of eating enough and being independent of total parenteral nutrition (TPN) or tube feeding as their ticket home from the hospital. Those patients were often classified as being the most resourceful and understanding of the importance of nutrition for their overall health status.

Barriers to good nutritional care include fasting before surgery and being transferred to other hospitals with loss of the opportunity to follow-up. Different cultural backgrounds or language barriers of patients were mentioned as other potential barriers. Older patients were identified as a group that could potentially have some challenges with dietary recording in the app, especially the elderly who are not used to smartphones or tablets. However, most of the interviewees thought the elderly would be able to use the app after a short introduction:

I even think that elderly persons who are not so fond of technical gadgets would have understood this, you know.

Nurse

### Inner Setting

The social architecture, age, maturity, and size of an organization constitute the structural characteristics construct [22]. Stability in the staff was described as being high. The 2 departments included in the study were organized differently. One was subdivided into groups, where each nurse belonged to one group taking care of patients in that specific group, whereas the physicians were rotating between the groups. This department also had group leaders organizing each of the groups, which was highlighted as being successful for the organization. The other department did not have any subdivision, and all nurses were potentially involved with all patients. The registered dietitians served the whole hospital, except for the children’s department.

The networks and communication construct involves the nature and quality of social networks and the formal and informal communication within an organization [22]. The middle managers of the department that was subdivided into groups reported that the communication and social networks were stronger within specific groups; however, they all recognized each other as colleagues.

There was uncertainty among the interviewees about the primary responsibility for nutritional care of patients. The majority described nurses as having the primary responsibility:

It’s mostly something the nurses try to talk about and assess. Ehm... but personally, I usually ask how things are going related to nutrition, and the nurses notify us how they [the patients] are doing with regard to food intake and digestion in general. [...] And we may contact the dietitian if we really need help, you know.

Physician

However, some respondents claimed that the physicians had formal responsibility for nutritional treatment, whereas the nurses took care of the day-to-day follow-up. Several nurses reported that they had to repeatedly remind the physicians about the prospect of tube feeding if the patient had no intake or very low intake.

The cooperation among nurses, physicians, and registered dietitians on the nutritional care of patients was, however, described as good in most cases. A diverging focus among the different groups of health care professionals was described among some of the nurses. This difference could potentially be confusing for patients:

It’s like, the physiotherapists are concerned about one thing [eg, do not drink juice because of coughing and possibility to get it in the lungs], and the dietitians are concerned about another thing [eg, eating enough protein]. We [the nurses] try to keep the threads together, and then the others are never satisfied.

Nurse

Culture includes norms, values, and basic assumptions of a given organization [22]. The majority of the health care professionals expressed that nutrition has an important role in the overall course of the disease for the patient, but the middle managers reported that they had no culture for specific routines related to screening for malnutrition or nutritional care:

Maybe it’s kind of based on what you feel, I don’t think it’s like it’s done the same way for all... [...] I guess we aren’t good enough to add something extra to the food or think about whole fat milk instead of fat-reduced milk, butter instead of... I guess it’s like... there’s no system I think. We could get very much better.

Middle manager

However, a positive shift with increased focus on nutrition had occurred recently. This included increased monitoring of food intake among malnourished or at-risk patients, use of medical nutrition drinks, and availability of food service hosts in the department’s buffet kitchen. Whereas an increased focus on the importance of nutrition was mentioned by several respondents, others reported on the challenges still present, especially among the physicians:

The physicians, the surgeons, are often pretty far away from recognizing nutrition as part of the whole. So I guess that’s a group who are a little more narrow-minded than the other physicians. But, it’s understandable. When you are so highly specialized you focus on your thing.

Registered dietitian

Tension for change is the degree to which stakeholders perceive the current situation as intolerable or needing change [22]. A general tension for change with regard to screening for malnutrition risk, monitoring, and treatment was highlighted by all groups of health care professionals. A perception among nurses was that they should probably have taken action with regard to nutrition earlier:

I wonder how many times I have heard like “oh, but I have several kilos to take away, so it’s no problem, it doesn’t matter if I don’t eat.” Because they are used to the disease passing away after a week when they are sick, and then there is no big deal because I eat when I get better. So I think we... Maybe you let it go too far before we start pushing that food, you know.

Nurse

The respondents were positive about the MyFood intervention because they wanted better tools for dietary assessment and follow-up. The nurses find the paper-based dietary recording forms used today to be time-consuming and unprecise. Uncertainty regarding the purpose of using the dietary recording forms existed. For some patients, dietary recording forms were used to identify the amount the patients could eat by themselves to supplement the remaining nutritional requirement through TPN or tube feeding. In some cases, the registered dietitians used the form to create a nutrition care plan. Several respondents described that the number of calories calculated from the forms was noted in the patient’s EPR, whereas others reported that this was only done in rare cases. They reported that the nurses working night shifts were supposed to perform the calorie calculations of the forms, but the compliance varied:

[...] I experience many patients having a dietary recording form lying on their nightstand that no one really... that has been lying there for several days, you know. And when the night shift replaces the form it’s like “Oh, this is from last week.”

Nurse

The readiness for implementation construct describes tangible and immediate indicators of organizational commitment to its decision to implement an intervention, including leadership engagement and available resources [22]. Leadership engagement is the commitment, involvement, and accountability of leaders and managers regarding the implementation [22]. Nutrition was described as having low priority in hospital management. None of the nurses or physicians was told by the management that nutrition should be prioritized:

Ehm... very seldom [signals from the management of nutrition focus]. This [nutrition] is seldom an issue from the management; at least as I have noticed.

Physician

A similar opinion was expressed by the middle managers. Nutrition was not a particular focus of the departments. The middle managers did not experience challenges regarding nutrition in their position between the nurses and top management:

No, I really think it works fine. Nutrition has so far been kind of a thing like anything else. It’s something we’re aware of, but maybe not enough. It constitutes a small part of all the challenges our patients have. But now that more focus has been set on nutrition, I feel that it’s established in all parts. That the nurses are positive about it and also those above me.

Middle manager

Available resources are the level of resources dedicated for implementation and ongoing operations, including finance, training, education, physical space, and time [22]. A concern regarding the availability of computers to check the reports in the MyFood system and read through recommended measures was raised. Some nurses mentioned a lack of time as a potential barrier:

The only thing I can think of is of course time, you know. Because that’s often a challenge in everything we do and all focus areas we’re supposed to have.

Nurse

Others expressed that the MyFood system would be a time saver:

This would have saved us a lot of time–not having to do that calculation [manual calculation of nutritional content] yourself.

Nurse

### Characteristics of Individuals

Knowledge and beliefs about the intervention involve individuals’ attitudes and the value placed on the intervention as well as familiarity with facts, truths, and principles related to the intervention [22]. The health care professionals expressed, in general, a positive attitude toward the MyFood intervention and saw several potential advantages related to the system compared with the current practice. Self-efficacy is the individuals’ belief in their own capabilities to execute courses of action to achieve implementation goals. The health care professionals expressed that they believed they would be able to use and follow up with MyFood.

### Process

Planning is defined as the degree to which a scheme or method of behavior and tasks for implementing an intervention are developed in advance, with the consideration of the quality of those schemes or methods [22]. The interviewees were asked to elaborate on their thoughts on how to perform an intervention study in the departments, including how to engage the nurses to follow up on the intervention within their busy schedules. The importance of providing everyone with information and assigning responsibility was highlighted among the nurses. The lack of information and assignment of responsibility will potentially decrease motivation. As the nurses are shift workers, it might be challenging to reach all nurses:

I think it’s important to inform absolutely everyone. Because we work in triple turrets many don’t get information, especially those working night shifts, and then they don’t see the importance of it maybe, because they haven’t received the information we have gotten now. And that has a lot to do with motivation, because if you haven’t received information and don’t know why we are doing it, then no one cares. So I think it’s very important to inform absolutely everyone who is going to take part, you know.

Nurse

Concrete examples of suggestions received were communicating information during morning meetings, increasing the night shift by an extra 30 min at the end of the shift, or requiring the nurses to arrive half an hour before the shift to reach all nurses working on all 3 shifts. Email communication was not recommended, as many nurses do not read their emails daily. Availability and daily visits to the department, including assistance and follow-up, were suggested. The possibility for nurses to call if they have questions was also recommended.

Engaging involves attracting and involving appropriate individuals in the implementation and use of the intervention through a combined strategy of social marketing, education, role modelling, training, and other similar activities [22]. Opinion leaders are individuals in an organization who have a formal or informal influence on the attitudes and beliefs of their colleagues with regard to implementing the intervention [22]. The physicians were described by some of the nurses as filling such an opinion leader role. However, not all nurses had this impression. Some claimed that some authorities among the nurses were more important as opinion leaders than the physicians. Group leaders and nurses with developmental responsibility were suggested as important to fill the position of implementation leaders. Nurses on the night shift were also suggested as key personnel, as they have the task of summarizing daily nutritional intake. Others expressed that a criterion for success was to assign the same responsibility to all nurses in the department. Group leaders and nurses with developmental responsibility were seen as potential champions to support and drive the implementation. Creating superusers with more experience who may inspire and help other nurses was also suggested.

## Discussion

### Principal Findings

This study used the CFIR framework [22] to identify current practices related to nutritional care in 2 departments in a university hospital in Norway. Perceived barriers and facilitators for the use of the MyFood system were assessed, and key aspects for an implementation plan were discussed. Screening for malnutrition risk was not prevalent or established as a routine in these departments. Dietary assessment and monitoring varied, as the nurses considered current procedures as being time- and resource-demanding. The use of the MyFood system was perceived as easier, more trustworthy, precise, fun, timesaving, and potentially facilitating increased awareness and implementation of nutritional treatment compared with the current practice. Cultural and language barriers, age of the patient, hygiene, availability of computers, time, and lack of interaction with EPRs were identified as potential barriers for use.

### Current Nutritional Care Practices

To explore how the MyFood system may be utilized in a hospital setting, it was important to obtain information on the current practices related to nutritional care. Despite national and European guidelines for screening for malnutrition risk [12,13], this was not routinely performed in the 2 departments (CFIR: Culture). This corresponds with results from Eide et al [14]. A recent scoping review on the use of technology to identify hospital malnutrition revealed malnutrition in the acute hospital setting to largely be an unrecognized problem, owing to insufficient monitoring, identification, and assessment of malnourished patients [18]. We emphasized a general tension for changing nutritional care practice among all the health care professional groups investigated. For the purpose of planning the upcoming implementation and effect study in the MyFood project, it is important to be aware that screening for malnutrition risk is not routinely performed. In this study, some of the interviewees mentioned that a screening procedure was now being implemented as part of the Norwegian Patient Safety Program [28].

Dietary recording among patients at risk of malnutrition was performed to some extent, and all interviewees seemed to be aware of this practice. However, dietary recordings were seldom followed up and the forms were frequently forgotten on the patient’s nightstand. A recent study at Oslo University Hospital based on the nutritionDay survey identified that only 41% of patients at malnutrition risk received nutritional treatment [1]. Several challenges with the current practice of using paper-based forms were described. The nurses in this study found it difficult to calculate the patients’ intake of energy, and they described the hospital food lists as containing too few details. This is in line with previous findings in which Eide et al [14] identified nurses to be uncertain about how to evaluate nutritional status, estimate nutritional needs, and measure energy and nutrient intake among hospitalized patients. An Australian study showed that poor knowledge of the nutrition care processes among nondietetic staff was a barrier to nutritional care of elderly hospitalized patients [17]. A lack of knowledge on nutritional treatment and follow-up has been reported as an important barrier to nutritional care among physicians and nurses in Scandinavian hospitals [16,35].

We did not reveal significant differences in the responses between the different groups of health care professionals. As described in the results for the networks and communication construct, the physicians stated that the nutritional care of patients was the nurses’ responsibility, whereas several nurses described the physicians as having the primary, formal responsibility. Eide et al [14] found that nurses were frustrated about the physicians’ low involvement and engagement in nutritional care of the patients. They also identified that the support from physicians in nutritional care made it easier to prioritize nutrition. This corresponds to our finding of the physicians’ important role in implementing new tools. Some nurses described communication between disciplines as challenging when different types of health care professionals have conflicting views. A literature review on communication between physicians and nurses revealed that communication tends to be unclear and unprecise, delaying patient care and increasing medical errors [36].

### Facilitators for the Use of the MyFood System

The health care professionals were generally positive about the MyFood system and acknowledged that the evidence-based recommendations forming the basis of the tool [12,28] were acceptable, as described for the CFIR construct of evidence strength and quality. They perceived the tool as easy to use (CFIR: Complexity), having a user-friendly and intuitive design (CFIR: Design, quality, and packaging), and believed they would be able to use the tool (CFIR: Self-efficacy). They saw the tool as potentially time saving, more precise, and trustworthy compared with current practices, as it related to the CFIR constructs of relative advantage and knowledge and beliefs about the intervention. The perceptions of the preciseness and trustworthiness of the dietary recording function in the MyFood system seemed to be based on assumptions that everything recorded in the app would be correct. Self-reported dietary assessment methods are, however, often associated with errors. The memory of intake, lack of motivation to record over several days, ability to estimate portion sizes, and perceptions of socially desirable responses are well-known challenges in self-reported intake [37]. An evaluation of the dietary recording function in the MyFood app found that MyFood was relatively accurate in estimating the patients’ intake of energy, protein, liquids, food, and beverages [29].

The MyFood system was perceived as potentially more fun and motivational to use compared with the current practice. Studies among adolescents have shown that dietary assessment using technology is preferred over paper-based food recording because electronic methods are perceived as more fun and motivational [38,39]. A systematic review of electronic methods to record food intake described that seeing progress toward fulfilment of goals can be highly motivational [40].

### Barriers for the Use of the MyFood System

Although the health care professionals were positive about MyFood, several potential barriers were identified. MyFood was recognized as potentially time saving, but some also described time used to follow up as a barrier for use. A lack of automatic transfer to the EPR was described as another potential barrier, related to the adaptability construct. Lack of time and integration with the EPR were also found to be barriers in the implementation of the eHealth intervention *Choice* for symptom reporting into clinical practice in a Norwegian university hospital [41]. Another potential barrier linked to adaptability was hygiene aspects related to the use of tablet computers among patients.

Perceived barriers associated with the CFIR construct patient needs and resources concerned different languages and cultural backgrounds among the patients. The MyFood tool includes both icons and pictures that may overcome some language challenges. If the use of the MyFood system turns out to be effective, the inclusion of several languages may be considered in the future. With regard to cultural barriers and patients eating foods not included in the hospital’s assortment, the MyFood app includes the possibility to record intake manually using a description of food or beverages consumed. In the long run, a wider range of food items may be included in the system. Challenges related to hygiene aspects may be solved by using plastic covers around the tablets. Older age was reported as a potential barrier to the use of MyFood owing to an increased risk of cognitive deficits or low self-efficacy among the elderly. However, qualitative studies among older persons have demonstrated that elderly people are often positive about using tablets and eager to learn [42,43]. A recent study describing an app to inspire home-dwelling elderly at nutritional risk to eat healthy foods showed that the elderly found the app easy to use [44].

### Key Aspects for an Implementation Plan

The health care professionals were positive about performing an intervention study to test the MyFood system in their department (CFIR: Trialability). The results related to the CFIR construct planning, where the interviewees elaborated on their thoughts on how to perform the intervention study, follow up, and engage the nurses, will be particularly relevant for the creation of an implementation plan. Of interest for the implementation plan are also results from the CFIR constructs: structural characteristics, networks and communication, and available resources. Important elements may include ongoing training, local technical assistance, clinical supervision, educational materials, support, availability, establishing an implementation team, and organizing clinician implementation meetings. To engage potential users of MyFood and identify opinion leaders, the involvement of potential champions and early adopters may also be of importance. These findings are previously described as relevant implementation strategies in the Expert Recommendations for Implementing Change project [21,45]. An important finding in this study is that nutritional care has low priority in management (CFIR: Leadership engagement). Leadership support and engagement are crucial [46] for successful implementation [47] and strategies toward the leaders should be included in the implementation plan.

### Strengths and Limitations

The strengths of the study are the inclusion of different health care professions and middle managers to reveal several views. The majority of the respondents were nurses, as they were considered to be the most important group with regard to the day-to-day nutritional care of the patients. Saturation is often described as the basis for sample size in qualitative studies [48]. However, this might not be the most appropriate [49]. Malterud et al [50] describe information power as related to the specificity of experiences, knowledge, or properties among the participants included in the sample. In this study, the health care professionals were holding characteristics specific to the study aim, in light of their professions. The aims of this study were relatively narrow and precise. Information power indicates that the more information the sample holds, relevant to the actual study, the lower the number of participants needed [50]. On the basis of these criteria, we considered our sample size to be sufficient.

The focus groups were originally composed of nurses with the same level of work experience to ensure that everyone’s voice was heard and to enable the more inexperienced nurses to talk freely in a separate group that was not dominated by more experienced nurses. Owing to illness among some nurses on the days of the interviews, some adjustments had to be made to be able to perform the focus group discussion with a sufficient number of nurses. Interviewing patients may have strengthened this study. Therefore, the patients will be included in the planned study of the implementation and effect of using the MyFood system.

Using an existing framework within the field of implementation science is considered an important strength to better understand, describe, and identify factors that predict the likelihood of implementation success. The CFIR framework identified the importance of knowledge for designing an implementation plan [51].

This study was performed in the same departments where the planned implementation and effect study will take place, thereby providing a local identification of potential barriers and facilitators that may be crucial. However, conducting the study at only 2 departments in 1 hospital may imply that our results are not necessarily representative of other departments or hospitals. A limitation is that this study was performed before the implementation of the MyFood system. The impression of MyFood was therefore based on a demonstration and perceived barriers and facilitators for use. This perception does not necessarily correspond to the real barriers and facilitators experienced in practice.

Whether or not the MyFood or a similar system, at some point in time, will be included and implemented in the Norwegian health care system is yet to be determined. There is a shift in the health care system toward increased use of digital systems. A need for the development of information and communication technology tools to screen, assess needs, monitor food intake, create a nutrition care plan, and follow up on disease-related malnutrition is described in a report from the Norwegian National Council for Nutrition [52].

### Conclusions

This study identified several challenges in the nutritional care of hospitalized patients at malnutrition risk in 2 departments in a university hospital in Norway. The use of the decision support system MyFood was anticipated to have several advantages compared with the current practice with nutritional follow-up. The MyFood system was perceived as more precise, trustworthy, fun, and motivational than the current practice. However, cultural, language, age, and hygiene aspects were perceived as potential barriers. The identification of perceived barriers and facilitators will be used in the creation of a plan to implement the MyFood system in clinical practice in an implementation and effect study.

## Abbreviations

CFIR: Consolidated Framework for Implementation Research
eHealth: electronic health
EPR: electronic patient record
TPN: total parenteral nutrition
TSD: tjenester for sensitive data (services for sensitive data)
UiO: University of Oslo
